# The rising prevalence of type 2 diabetes among the youth in southern India—An ancillary analysis of the Secular TRends in DiabEtes in India (STRiDE‐I) study

**DOI:** 10.1111/1753-0407.13576

**Published:** 2024-06-26

**Authors:** Arun Nanditha, Priscilla Susairaj, Krishnamoorthy Satheesh, Arun Raghavan, Chamukuttan Snehalatha, Ambady Ramachandran

**Affiliations:** ^1^ India Diabetes Research Foundation and Dr. A. Ramachandran's Diabetes Hospitals Chennai India

**Keywords:** family history, incidence, obesity, prevalence, type 2 diabetes, young diabetes

## Abstract

**Background:**

We studied the prevalence and incidence of type 2 diabetes (T2DM) and its associated risk factors in younger (20 and 39 years) and older individuals (≥40 years) over a 10‐year period.

**Methods:**

Epidemiological surveys in 2006 (*n* = 7066) and 2016 (*n* = 9848) were conducted in similar urban and rural locations of southern India among people aged ≥20 years. Diagnosis of T2DM was made using World Health Organization criteria. Self‐reported diabetes was verified from medical records. Age and gender standardized prevalence and incidence rates, percentage change in obesity, hypertension, and dyslipidemia were calculated. Prevalence ratios (PR) were calculated using Poisson regression analyses. Primary study was registered on www.ClinicalTrials.gov. Identifier: NCT03490136.

**Results:**

In 10 years, the prevalence of T2DM increased in younger (7.8% vs. 4.5%, *p* < 0.0001) and older individuals (34% vs. 28.4%, *p* < 0.0001). After adjusting for age, family history of diabetes, and waist circumference, younger individuals showed a higher percentage increase in prevalence than the older group (PR = 1.36 [95% confidence interval [CI], 1.14–1.62], *p* = 0.001) versus (PR = 1.11 [95% CI, 1.02–1.20], *p* = 0.02). Increase in rates of obesity and dyslipidemia was also higher in the younger than in the older individuals. In 10 years, incidence of T2DM increased by 120% (1.1% vs. 0.5%, *p* < 0.0001) and 150% (5% vs. 2%, *p* < 0.0001) in the younger and older individuals, respectively.

**Conclusions:**

Higher percentage increase in prevalence of T2DM was seen among younger individuals over a 10‐year period. Obesity and family history of diabetes were shown to be the primary contributing factors for the rise in prevalence.

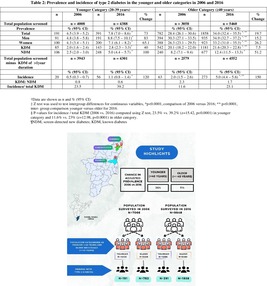

## Graphical abstract

Highlights

- The study reports a comparative profile on prevalence and incidence of T2DM and its associated risk factors in the younger and the older individuals in 2006 and 2016.
- Prevalence of T2DM increased both in the younger (36%) and older individuals (11%) in a decade's time.
- Incidence rose by 120% in the younger individuals and 150% in the older group.
- Percentage increase in cardiometabolic risk was higher among the younger than in the older individuals.
- Obesity and family history of diabetes were the primary contributing factors for the rising prevalence in both groups.

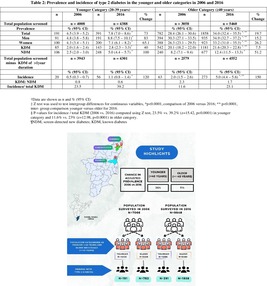

## 1 INTRODUCTION

The diabetes epidemic in India showed a sharp increase, from 33 million people in the year 2000 to 72 million people in 2021. This is further set to reach a 125 million by 2045.^1^ Although increasing age of the population and lower mortality rates are the primary factors for the disease burden, recent studies show a steady decline in the mean age of individuals with type 2 diabetes (T2DM).^2^, ^3^, ^4^ The South Asian Indians with their inherent characteristics defined as the thin‐fat phenotype together with the adverse environmental influences make them more vulnerable to developing hyperglycemia at a relatively young age than the White populations.^5^, ^6^

In our earlier study, we observed that in a 10‐year period, the prevalence of T2DM increased from 18.3% to 22.5% and prediabetes from 13.5% to 21.5% in the age group of 35–44 years.^7^ The impact of T2DM among the younger population is heightened by increased risk of early complications, requirement for intensive management of diabetes, and adverse psychological factors affecting the productive years of life.^4^ According to an all India register for youth‐onset diabetes, more than 25% were diagnosed with T2DM at <25 years of age; incidence was 0.5 cases/100 000 under 19 years.^8^, ^9^ Based on the exposure of the population to risk factors, an analysis using the Markov simulation model showed that the lifetime risk for diabetes of a 20‐year old man and woman were 55.5% (95% confidence interval [CI], 51.6–59.7) and 64.6% (95% CI, 60–69.9), respectively. Lifetime risk declined with age; at 60 years, the risk was 37.7% (95% CI, 30.1–46.7) in women and 27.5% (95% CI, 23.1–32.4) in men.^10^

Recent studies from India that have compared the clinical, metabolic, and treatment profile of patients diagnosed below 40 years were primarily hospital based or from tertiary care centers.^11^, ^12^ To understand the scenario at a regional level, we aimed to study the prevalence of T2DM and its associated risk factors among the younger (20–39 years) and older individuals (≥40 years) at two time points using the population data of 2006 and 2016.^7^ The secondary objectives were to estimate the incidence of T2DM and the prevalence of cardiometabolic factors in the younger and older age categories.

## 2 MATERIALS AND METHODS

### 2.1 Study population

This analysis was performed using the data of two epidemiological surveys conducted in 2006 and 2016 in three distinct geographical regions; a city (Chennai), town (Kanchipuram, 80 km from Chennai), and peri‐urban villages (Panruti, 186 km from Chennai) in southern India. According to the Indian census commission, a city has an urban population of over 4 million; a town is smaller than a city with a minimum population of 100 000 with at least 75% men engaged in nonagricultural work. Peri‐urban villages (PUVs) are landscapes between a town and rural area where people are predominantly agricultural or casual laborers. Survey‐I was conducted between February and June 2006 and survey‐II from July 2016 to September 2017. The two surveys, done 10 years apart, were conducted by the research team of India Diabetes Research Foundation, Chennai. Methodologies for sample selection, screening, anthropometric measurements, biochemical estimations, and assessment of behavioral aspects were similar in both surveys.^7^, ^13^

A multistage random selection was done from streets with similar population characteristics in 2006 and 2016 for an unbiased representation of all socioeconomic strata. In the city, five corporation zones were randomly selected; in the town, municipal wards and in the PUV, panchayat census wards were used. In the PUV, 18 villages with population ranging from 2054 to 83l3 persons were selected. Enumeration and screening were done by house to house visits. All eligible persons in a household (≥20 years) were invited for screening. Enrollment and study assessments were performed after the participants had given a written informed consent.

#### 2.1.1 Age categorization

Individuals with a diagnosis of T2DM between 20 and 39 years were grouped under younger category and those with a diagnosis at ≥40 years in the older category. Individuals with type 1 diabetes or other forms of diabetes were excluded. Presence of type 1 diabetes was ruled out by a physician during medical examination, reference to past medical history and by signs and symptoms.

### 2.2 Anthropometry and vital signs

Height, weight, and waist circumference (WC) were measured by standard procedures.^13^ Persons with body mass index (BMI) (weight in kg/height in m^2^) of ≥23 kg/m^2^ were considered as having generalized obesity (GO).^14^ Abdominal obesity (AO) was indicated by WC of ≥90 cm for men and ≥80 cm for women.^14^

Blood pressure was measured in the sitting position using the electronic Omron machine (Omron Corporation, Tokyo, Japan). An average of two readings taken at 5‐min interval was recorded. Self‐reported history of hypertension (HTN) with details of medication was noted. Participants with a history of HTN and newly diagnosed cases with blood pressure readings ≥140/90 mmHg were categorized as hypertensive.^15^

### 2.3 Biochemical assessments

Estimations of plasma glucose were done after 8–12 h of overnight fast and at 2 h post 75 g of oral glucose load. In known cases of T2DM, fasting plasma glucose (FPG) and 2‐h postprandial glucose were performed. Glucose estimations were performed using venous blood with Accuchek Performa (Roche Diagnostics, GmbH, Germany) by the hexokinase method and calibrated to read plasma glucose values.^16^ We calculated the correlation between plasma glucose tested in the venous blood by glucometer and plasma glucose estimated in the laboratory in 111 subjects; plasma glucose = 17.1 + 0.887 × venous plasma glucose (glucometer), *p* < 0.0001. A diagnosis of T2DM was made if FPG was ≥7.0 mmol/L (≥126 mg/dL) and/or a 2 h postglucose ≥11.1 mmol/L (≥200 mg/dL), using the World Health Organization criteria.^17^ Fasting serum lipids were estimated by standard enzymatic procedures (Roche Diagnostics, Germany). High‐density lipoprotein cholesterol (HDLc) was estimated by the direct assay method. The autoanalyzer was calibrated for each parameter at the start of the estimations. If variations of >10% were seen, calibration was repeated. Limits of intra‐assay and inter‐assay coefficients of variation were <10% and <12%, respectively. Quality control was performed at internals of 25 samples with standard controls. External performance monitoring was conducted by Bio‐Rad.

### 2.4 Medical history

Self‐reported diabetes was noted with details of diagnosis and treatment, verified from medical records or prescriptions given by the treating physicians. Duration of diabetes could be verified for 85% of the self‐reported cases in the city and town and for 53% in the rural areas. In the cases used for calculation of incidence (<1 year duration) in the city and town, more than 90% of the self‐reported duration could be verified from the medical records or date of prescription. In the villages, this was possible in 65%. In 2006, during the verification, 4% of cases had to be changed from <1 year to more than 1 year in the urban population and in 5.2% in the rural population. In 2016, the respective numbers were 2.5% in the urban and 4.6% in the rural population. We did not have to change any case from >1 year to <1 year. Screen‐detected cases were denoted as new diabetes (NDM) and self‐reported cases as known diabetes (KDM).

### 2.5 Lifestyle behavior

Nutrient intake was recorded by trained dietitians using the 24‐h dietary recall method. The total energy intake (kcal) and components of individual food constituents (carbohydrates, proteins and fat [in grams]) were calculated with an in‐house dietary analysis program (visual basic programming tool) using the National Institute of Nutrition guidelines for India.^18^ Total calorie consumption was divided as normal calorie intake (33rd percentile), moderately high calorie intake (66th percentile), and very high calorie intake (99th percentile). Details of physical activity were recorded and quantified using a questionnaire. The quantification was based on the occupational activity, hours of moderate/ vigorous activity, and leisure time activity. A score of 7–70 was used. The questionnaires used were validated in our previous studies.^13^, ^19^

### 2.6 Statistical analyses

#### 2.6.1 Sample size calculation

In survey‐I, the required sample for Chennai (*n* = 1712) was calculated with an assumed increase in prevalence, since earlier surveys, from 13.5% to 17%; for Kanchipuram (*n* = 1422), from 7% to 10%, and Panruti (*n* = 1275) from 6% to 9%.^20^, ^21^, ^22^ A total of 8216 people were invited for screening and 7066 were enrolled. In survey‐II, the required sample size for Chennai was 3824, for an increase in prevalence from 18.6% to 21.0%. For the town (*n* = 3547) and PUV (*n* = 2434), the calculation was based on anticipated increases from 16.4% to 19% and from 9.2% to 12.2%, respectively.^13^ In both surveys I and II, the sample size was calculated at 80% power with an alpha error of 5%. In survey‐II, a total of 11 191 people were invited for screening, and 9848 were enrolled. The demographic characteristics of the responders and nonresponders were similar in both surveys.

#### 2.6.2 Study analyses

Data are presented as mean and standard deviation for continuous variables and as proportion for categorical variables. For normally distributed variables, intergroup comparisons by the independent sample “*t*” test and *Z* test were used to compare categorical variables.

Prevalence was calculated as the number of persons with diabetes (NDM + KDM) among the total number of adults screened. The duration of diabetes for each case of KDM was calculated by subtracting the age at diagnosis from the age at the time of the interview. Incidence was calculated as the percentage of survey participants with a self‐reported history of known diabetes (KDM) with ≤1 year of duration (numerator) among the total survey population minus the number of KDM cases with >1 year of duration (denominator). A similar method had been used in earlier studies to calculate the incidence.^23^ The ratio of KDM to NDM was also calculated to assess the change in the rate of clinical detection of diabetes. Estimates of prevalence of diabetes were age and sex standardized by a direct standardization method using the 2001 and 2011 census data for the respective populations in Tamil Nadu.

To study the secular change in metabolic abnormalities in 10 years, percentage differences of GO, AO, HTN, and DL between the years 2006 and 2016 were computed. Dyslipidemia (DL) was defined as one or more of the following lipid abnormalities: total cholesterol ≥5.2 mmol/L, triglycerides ≥1.7 mmol/L, and total cholesterol:HDLc ratio ≥4.5.

The prevalence ratios (PRs) (2016 vs. 2006) of diabetes were calculated using Poisson regression analyses with robust variance to identify the risk variables associated with T2DM versus nondiabetes (dependent variable) in the two age categories. Independent variables included in the analyses were study years 2016 versus 2006, age (units of 10), WC (units of 10), total calories (reference: normal calorie intake), hypertension (reference: no), rural habitat (reference: no), gender (reference: female), physical activity (reference: activity), education (reference: educated), and occupation (reference: nonlaborer). The unadjusted PR was calculated for the year 2016 versus 2006. Variables identified by logistic regression analysis separately for 2006 and 2016 were included in the analysis of PR. The variables were adjusted in a step‐wise manner to study the influence of each risk factor on the PR for the year 2016 versus 2006 in the two age categories. The statistical package SPSS, version 21.0 was used for analyses.

#### 2.6.3 Ethical approval

The primary study was registered on www.ClinicalTrials.gov. Identifier: NCT03490136. The study was approved by the Ethics Committee of the India Diabetes Research Foundation and Dr. A. Ramachandran's Diabetes Hospitals. An independent data monitoring committee reviewed the progress of the survey.

## 3 RESULTS

**FIGURE 1:**
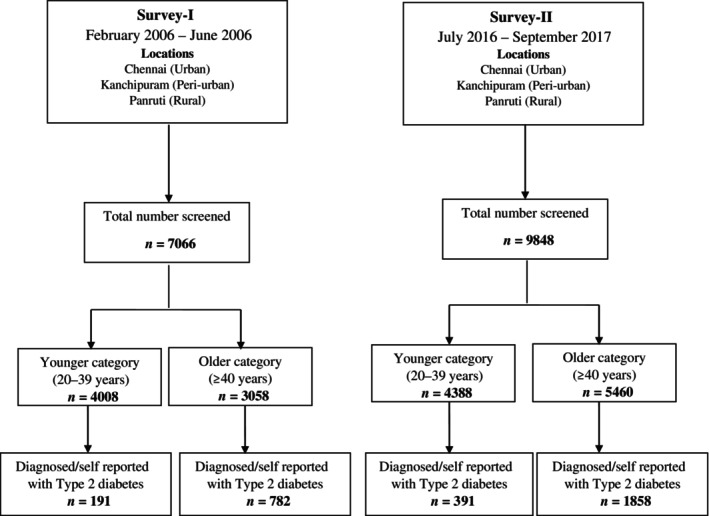
Details of sampling procedure in the surveys conducted in 2006 and 2016.

The analysis was carried out using data of survey‐I (*n* = 7066) and survey‐II (*n* = 9848) conducted during 2006 and 2016, respectively (Figure 1). The participants in the two surveys were categorized as younger (20–39 years) and older (≥40 years) individuals. Table 1 shows the change in the general characteristics and cardiovascular risk factors over 10 years' time in the younger and older individuals. A comparison between the two categories for the years 2006 and 2016 is also shown.

**TABLE 1:** Characteristics of the surveyed population in the younger and older categories in the years 2006 and 2016.

	Younger category (20–39 years)			Older category (≥40 years)		
	2006	2016	*p* value	2006	2016	*p* value
	(*n* = 4008)	(*n* = 4388)		(*n* = 3058)	(*n* = 5460)	
Family history, *n* (%)	817 (20.4)*	1178 (26.8)*	<0.0001	539 (17.6)	1277 (23.4)	<0.0001
Male:female	1919:2089	1884:2504		1402:1656	2643:2817	
Age (years)	29 ± 5	30 ± 5	<0.0001	49 ± 7*	51 ± 8*	<0.0001
Anthropometry						
BMI (kg/m^2^)	22.5 ± 4.6	24.5 ± 4.8	<0.0001	23.8 ± 4.7*	25.1 ± 4.7*	<0.0001
WC, men (cm)	79 ± 10	84 ± 11	<0.0001	84 ± 10*	88 ± 11*	<0.0001
WC, women (cm)	78 ± 10	83 ± 11	<0.0001	82 ± 11*	86 ± 10*	<0.0001
Vitals						
SBP (mmHg)	117 ± 11	114 ± 14	<0.0001	122 ± 13*	126 ± 19*	<0.0001
DBP (mmHg)	75 ± 8	77 ± 10	<0.0001	77 ± 9*	82 ± 11*	<0.0001
Diet						
Calories (kcal)	1960 ± 338	2134 ± 195	<0.0001	1951 ± 327	2131 ± 190	<0.0001
Carbohydrate (%)	67 ± 4	66 ± 4	<0.0001	67 ± 4*	66 ± 4	<0.0001
Protein (%)	10 ± 1	11 ± 1	<0.0001	10 ± 1	11 ± 1*	<0.0001
Fat (%)	20 ± 4	22 ± 4	<0.0001	20 ± 4	21 ± 4	<0.0001
Physical activity						
Sedentary, *n* (%)	789 (19.7)*	1116 (25.4)*	<0.0001	543 (17.8)	1079 (19.8)	0.024
Light, *n* (%)	1635 (40.8)	1763 (40.2)	0.575	1308 (42.8)	2451 (44.9)*	0.061
Moderate, *n* (%)	538 (13.4)	686 (15.6)	0.004	544 (17.8)*	960 (17.6)*	0.816
Heavy, *n* (%)	1046 (26.1)*	823 (18.8)	<0.0001	663 (21.7)	970 (17.8)	<0.0001

			*p* value	% Change			*p* value	% Change
Generalized obesity [BMI ≥23 (kg/m^2^)], *n* (%)	1669 (41.6)	2615 (59.6)	<0.0001	43.3	1692 (55.3)	3583 (65.6)**	<0.0001	18.6
Abdominal obesity in men [WC ≥90 (cm)], *n* (%)	289 (14.9)	625 (33.2)	<0.0001	122.8	455 (32.5)	1194 (45.2)**	<0.0001	39.1
Abdominal obesity in women [WC ≥80 (cm)], *n* (%)	962 (46.1)	1560 (62.3)	<0.0001	35.1	987 (59.6)	2114 (75.0)**	<0.0001	25.8
Hypertension, *n* (%)	406 (10.1)	563 (12.8)	<0.0001	26.7	823 (26.9)	1947 (35.7)**	<0.0001	32.7
Dyslipidemia, *n* (%)	2708 (67.6)	3121 (71.1)	<0.0001	5.2	2261 (73.9)	4062 (74.4)**	<0.0001	0.7

*Note*: Data are presented as mean ± SD for continuous variables and as *n* (%) for categorical variables.

Abbreviations: BMI, body mass index; DBP, diastolic blood pressure; SBP, systolic blood pressure; SD, standard deviation; WC, waist circumference.

Intergroup comparison, *p < 0.05, younger versus older for 2006 and 2016 using independent sample “*t*” test for mean ± SD and *Z* test for *n* (%).

** *p* < 0.05, comparison of younger versus older for 2016.

At both time points, a greater proportion of the younger individuals had a family history of diabetes than the older persons. Body mass index, WC, SBP, and DBP were higher in the older than in younger group (*p* < 0.0001 for all) in both surveys. Except for SBP in the younger category, all anthropometric variables and DBP increased significantly in the population over the time.

In both surveys, dietary consumption was similar in the both categories. Among the individual dietary components, total calorie intake, protein, and fats increased over the 10 years in the population (*p* < 0.0001 for all). The proportion involved in sedentary activity increased in both categories; it was comparatively higher in younger than in older individuals at both time points. In 2016, the proportion having heavy physical activity decreased in both; moderate activity increased only among the younger individuals.

Presence of cardiovascular risk factors viz., GO, AO (in men and women), HTN, and DL were higher in the older than in the younger group (*p* < 0.0001 for all). Over the 10‐year period, the percentage increase in these risk factors was seen in both categories; it was comparitively higher in the younger than in the older persons.

Table 2 shows the prevalence and incidence of T2DM in the younger and older individuals in the years 2006 and 2016. In both surveys, men had a higher prevalence of diabetes than women. Between 2006 and 2016, the prevalence and incidence of T2DM increased significantly in both age categories. The percentage increase in prevalence was greater in the younger (73%) than in the older persons (19.7%).

**TABLE 2:** Prevalence and incidence of type 2 diabetes in the younger and older categories in 2006 and 2016.

	Younger category (20–39 years)					Older category (≥40 years)				
	*n*	2006	*n*	2016	% Change	*n*	2006	*n*	2016	% Change
Total population screened		*n* = 4008		*n* = 4388			*n* = 3058		*n* = 5460	
Prevalence		% (95% CI)		% (95% CI)			% (95% CI)		% (95% CI)	
Total	191	4.5 (3.9–5.2)	391	7.8 (7.0–8.6)*	73	782	28.4 (26.1–30.6)	1858	34.0 (32.4–35.5)*, **	19.7
Men	91	4.8 (3.8–5.8)	191	8.8 (7.5–10.1)*	83	394	30.3 (27.1–33.5)	935	34.9 (32.7–37.2)*, **	15.2
Women	100	4.3 (3.4–5.1)	200	7.1 (6.1–8.2)*	65.1	388	26.3 (23.1–29.5)	923	33.2 (31.0–35.3)*, **	26.2
KDM	85	2.0 (1.6–2.4)	143	2.8 (2.3–3.3)*	40	542	20.1 (18.2–22.0)	1181	21.6 (20.3–22.8)*, **	7.5
NDM	106	2.5 (2.0–3.0)	248	5.0 (4.4–5.7)*	100	240	8.2 (7.1–9.4)	677	12.4 (11.5–13.3)*	51.2

Total population screened minus KDM of >1‐year duration		*n* = 3943		*n* = 4301			*n* = 2579		*n* = 4552	
		% (95% CI)		% (95% CI)			% (95% CI)		% (95% CI)	
Incidence	20	0.5 (0.3–0.7)	56	1.1 (0.8–1.4)*	120	63	2.0 (1.5–2.6)	273	5.0 (4.4–5.6)*, **	150
KDM:NDM		0.8		0.6			2.3		1.7	
Incidence/total KDM		23.5		39.2			11.6		23.1	

*Note*: Data are shown as *n* and % (95% CI). *Z* test was used to test intergroup differences for continuous variables, *p*‐values for incidence/total KDM (2006 vs. 2016) computed using *Z* test, 23.5% versus 39.2% (*z* = 15.42, *p* < 0.0001) in younger category and 11.6% versus 23% (*z* = 12.98, *p* < 0.0001) in older category.

Abbreviations: CI, confidence interval; KDM, known diabetes; NDM, screen‐detected new diabetes.

* *p* < 0.0001, comparison of 2006 versus 2016;

** *p* < 0.0001, intergroup comparison younger versus older for 2016.

In a 10‐year period, the incidence of T2DM increased among the young by 120% (0.5% [95% confidence interval (CI), 0.3–0.7] vs.1.1% [95% CI, 0.8–1.4], *p* < 0.0001) and by 150% (2% [95% CI, 1.5–2.6] vs. 5% [95% CI, 4.4–5.6], *p* < 0.0001) among the older individuals. The ratio of KDM to NDM decreased in both age groups. Data from the two time points show that the proportion of incident diabetes to total KDM rose from 23.5% to 39.2% (*p* < 0.0001) in the younger and from 11.6% to 23% in the older individuals (*p* < 0.0001).

Table 3 shows the profile of other cardiovascular risk factors in the younger and the older individuals with T2DM in the years 2006 and 2016. In both surveys, family history of T2DM was reported more among the younger than the older individuals. Over a 10‐year period, BMI, WC, and DBP increased in both categories (*p* < 0.0001), whereas SBP increased only among the older group (*p* < 0.0001). In 2016, the younger group had a relatively higher BMI than the older individuals (27.0 ± 4.9 vs. 26.1 ± 4.6, *p* < 0.001). No significant differences in total calorie intake and individual dietary components were seen between the categories. In the survey year 2016, intake of protein and fat increased in both groups (*p* < 0.0001 for all components). Significant differences in physical activity were reported only in the younger individuals between the time points. The proportion involved in moderate (*p* < 0.0001) to heavy activity (*p* < 0.0001) decreased, whereas those involved in sedentary (*p* < 0.0001) to light activity (*p* < 0.01) increased. In 2006, the proportion involved in sedentary activity was more in the older group (26.9% vs. 16.2%, *p* = 0.002); a higher proportion of younger individuals reported heavy activity (26.2% vs. 18.2%, *p* = 0.013). In 2016, no significant differences were observed between the two age categories.

**TABLE 3:** Profile of type 2 diabetes (T2DM) and other cardiovascular risk factors in the younger and older individuals in 2006 and 2016.

	Younger category (20–39 years)			Older category (≥40 years)		
	2006 (*n* = 191)	2016 (*n* = 391)	*p* value	2006 (*n* = 782)	2016 (*n* = 1858)	*p* value
Family history, *n* (%)	75 (39.3)*	162 (41.4)*	0.629	246 (31.5)	561 (30.2)	0.508
Male:female	91:100	191:200	—	394:388	935:923	—
Age (years)	32 ± 5	34 ± 4	0.004	52 ± 8	53 ± 8	<0.0001
Anthropometry						
BMI (kg/m^2^)	25.3 ± 4.4	27.0 ± 4.9*	<0.0001	25.2 ± 4.3	26.1 ± 4.6	<0.0001
WC, men (cm)	85 ± 10	90 ± 10	<0.0001	88 ± 10	91 ± 10	<0.0001
WC, women (cm)	85 ± 10	90 ± 11	<0.0001	87 ± 10	90 ± 9	<0.0001
Vitals						
SBP (mmHg)	122 ± 12	122 ± 14	0.942	127 ± 14	131 ± 19*	<0.0001
DBP (mmHg)	77 ± 8	83 ± 9	<0.0001	80 ± 9*	84 ± 11	<0.0001
Diet						
Calories (kcal)	2036 ± 309	2139 ± 184	<0.0001	1938 ± 317	2140 ± 188	<0.0001
Carbohydrate (%)	67 ± 4	65 ± 4	<0.0001	66 ± 4	65 ± 4	<0.0001
Protein (%)	10 ± 1	11 ± 1	<0.0001	10 ± 1	11 ± 1	<0.0001
Fat (%)	20 ± 4	22 ± 4	<0.0001	21 ± 4	22 ± 4	<0.0001
Physical activity						
Sedentary, *n* (%)	31 (16.2)	97 (24.8)	<0.0001	210 (26.9)*	450 (24.2)	0.144
Light, *n* (%)	77 (40.3)	173 (44.2)	0.0003	305 (39.0)	782 (42.1)	0.140
Moderate, *n* (%)	33 (17.3)	54 (13.8)	<0.0001	125 (16.0)	300 (16.1)	0.949
Heavy, *n* (%)	50 (26.2)*	67 (17.1)	<0.0001	142 (18.2)	326 (17.5)	0.667

			*p* value	% change			*p* value	% change
Generalized obesity [BMI ≥23 (kg/m^2^)]	133 (69.6)	314 (80.3)**	<0.0001	15.4	545 (69.7)	1403 (75.5)	<0.0001	8.3
Abdominal obesity in men [WC ≥90 (cm)]	29 (31.9)	107 (56.0)	<0.0001	75.6	174 (44.2)	530 (56.7)	<0.0001	28.3
Abdominal obesity in women [WC ≥80 (cm)]	74 (74.0)	173 (86.5)	<0.0001	16.9	300 (77.3)	798 (86.5)	<0.0001	11.9
Hypertension	43 (22.5)	109 (27.9)	<0.0001	24	325 (41.6)	895 (48.2)**	<0.0001	15.9
Dyslipidemia	149 (78.0)	336 (85.9)**	<0.0001	10.1	632 (80.8)	1514 (81.5)	<0.0001	0.9

*Note*: Data are presented as mean ± SD for continuous variables and as *n* (%) for categorical variables.

Abbreviations: BMI, body mass index; DBP, diastolic blood pressure; SBP, systolic blood pressure; SD, standard deviation; WC, waist circumference.

Intergroup comparison, *p < 0.05, younger versus older for 2006 and 2016 using independent sample “*t*” test for mean ± SD and *Z* test for *n* (%);

** *p* < 0.05, comparison of younger versus older for 2016.

Both categories showed an increase in GO, AO, and HTN in 2016 (*p* < 0.0001 for all). Increase in DL was noted in the younger group (*p* < 0.0001), whereas it remained unchanged in the older group. Over the 10 years, a higher percentage of increase in GO, AO, HTN, and DL were seen in the younger than in the older individuals.

Table 4 shows that the unadjusted prevalence of T2DM in the younger individuals increased by 87% (PR = 1.87 [95% CI, 1.57–2.22], *p* < 0.0001) and by 33% in the older group (PR = 1.33 [95% CI, 1.22–1.45], *p* < 0.0001). In a step‐wise approach, the addition of age, family history of T2DM, and WC, each reduced the PR (i.e., explained a portion of the increase in prevalence between 2006 and 2016), collectively reducing the PR for T2DM to 1.36 (95% CI, 1.14–1.62), *p* = 0.001 and 1.11 (95% CI, 1.02–1.20), *p* = 0.02 in the younger and older categories, respectively. Further addition of HTN to the model did not modify the PR further. Rural habitat and calorie intake were not included for the younger category as they showed no association with T2DM. The survey year effect was nullified by addition of these factors for the older category.

**TABLE 4:** Prevalence ratio for type 2 diabetes (T2DM) between 2016 and 2006 in the younger and older categories: results of Poisson regression analyses.

	Younger category (20–39 years)		Older category (≥40 years)	
	Exp (β) (95% CI)	*p* value	Exp (β) (95% CI)	*p* value
Unadjusted				
2016 versus 2006	1.87 (1.57–2.23)	<0.0001	1.33 (1.22–1.45)	<0.0001
Adjusted				
Model 1				
2016 versus 2006	1.65 (1.39–1.97)	<0.0001	1.26 (1.16–1.37)	<0.0001
Age (years, units of 10)	3.38 (2.74–4.17)	<0.0001	1.34 (1.28–1.41)	<0.0001
Model 2				
2016 versus 2006	1.58 (1.33–1.88)	<0.0001	1.21 (1.11–1.32)	<0.0001
Age (years, units of 10)	3.27 (2.65–4.03)	<0.0001	1.41 (1.34–1.48)	<0.0001
Family history of diabetes (yes)	1.99 (1.69–2.35)	<0.0001	1.75 (1.61–1.90)	<0.0001
Model 3				
2016 versus 2006	1.36 (1.14–1.62)	0.001	1.11 (1.02–1.20)	0.02
Age (years, units of 10)	2.76 (2.24–3.41)	<0.0001	1.39 (1.32–1.46)	<0.0001
Family history of diabetes (yes)	1.69 (1.43–1.99)	<0.0001	1.59 (1.46–1.73)	<0.0001
WC (cm, units of 10)	1.46 (1.36–1.56)	<0.0001	1.30 (1.25–1.34)	<0.0001
Model 4				
2016 versus 2006	1.35 (1.13–1.61)	0.001	1.09 (1.00–1.19)	0.05
Age (years, units of 10)	2.64 (2.14–3.27)	<0.0001	1.33 (1.27–1.40)	<0.0001
Family history of diabetes (yes)	1.65 (1.39–1.96)	<0.0001	1.56 (1.44–1.70)	<0.0001
WC (cm, units of 10)	1.40 (1.30–1.51)	<0.0001	1.26 (1.22–1.30)	<0.0001
Hypertension (yes)	1.71 (1.41–2.07)	<0.0001	1.42 (1.32–1.54)	<0.0001

*Note*: Factors significantly associated with T2DM in the younger and older category in the logistic regression analysis were included in the Poisson regression equation. Younger category: 2016 versus 2006, age, family history of diabetes, waist circumference (WC), hypertension. Older category: 2016 versus 2006, age, family history of diabetes, waist circumference, hypertension, rural habitat, calories (moderately high and very high intake). Dependent variable: diabetes versus nondiabetes. Independent variables added as per the model given below. Model 1: Study years 2016 versus 2006 + age (units of 10). Model 2: Study years 2016 versus 2006 + age (units of 10) + family history of diabetes (yes). Model 3: Study years 2016 versus 2006 + age (units of 10) + family history of diabetes (yes) + WC (units of 10). Model 4: Study years 2016 versus 2006 + age (units of 10) + family history of diabetes (yes) + WC (units of 10) + hypertension (reference: no). Model 5: Study years 2016 versus 2006 + age (units of 10) + family history of diabetes (yes) + WC (units of 10) + hypertension (reference: no) + rural habitat (reference: no). Model 6: Study years 2016 versus 2006 + age (units of 10) + family history of diabetes (yes) + WC (units of 10) + hypertension (reference: no) + rural habitat (reference: no) + calories (reference: normal). Models 5 and 6 are not shown in Table 4, since rural habitat and calorie intake were not associated with T2DM in the younger category.

Abbreviation: CI, confidence interval.

## 4 DISCUSSION

This analysis of the Secular TRends in DiabEtes in India (STRiDE‐I) study shows the comparative profiles of T2DM and associated risk factors among the younger and older individuals in a 10 year period. Between the surveyed years, prevalence of T2DM increased both in the younger (4.5% vs. 7.8%, *p* < 0.0001) and the older persons (28.4% vs. 34%, *p* < 0.0001). Although prevalence was higher in the older than in the younger people (7.8% vs. 34%, *p* < 0.0001), after controlling for significant risk factors viz., age, positive family history of diabetes, and WC, the younger individuals showed a higher increase than their counterparts (36% vs. 11%). In addition, higher rates of increase in GO, AO and DL were seen in the younger than in the older individuals. Over a 10‐year period, incident cases increased in both groups: by 120% (1.1% vs. 0.5%, *p* < 0.0001) and 150% (5% vs.2%, *p* < 0.0001) in the younger and older persons, respectively.

In recent times, studies across varied ethnicities have reported increasing numbers of adults diagnosed with T2DM at age ≤40 years.^24^ A retrospective cohort study in the United Kingdom among newly diagnosed T2DM between 1990 and 2010 reported a major increase in persons aged ≤40 years at diagnosis.^25^ The incidence ratio (per 100 000 population) of newly diagnosed cases (≤40 years) increased from 217 in the years 1996–2000 to 598 in 2006–2010.^25^ A study in the United States showed higher estimates of T2DM in the South Asian, Black, or Hispanic ethnicity compared with the White populations.^26^ The Joint Asia Diabetes Evaluation (JADE)^27^ cohort that included data from nine countries or regions including India, Philippines, China, and Hong Kong showed that one in five patients visiting the clinic were diagnosed with T2DM at <40 years. The younger population had a mean age of 32.9 (±5.7) versus 53.9 (±9) in the older group.^27^

Although the underlying pathophysiology of T2DM appears similar in both categories, presence of certain risk factors tends to be associated with the development of the disease a decade earlier in the younger population. We observed a significantly greater proportion of the younger individuals to have a family history of diabetes than the older group in both the survey years. A probable reason could be that younger individuals have a definite recollection of their parents' health history than older persons. However, the risk due to familial aggregation and strong genetic predisposition were shown in early studies including our own, where individuals with parental history of diabetes or abnormal glucose tolerance had a younger age at onset.^28^, ^29^, ^30^, ^31^ An earlier report including Asian Indians, Australian Aboriginals, and Pacific Islanders showed that age at onset decreased by 1.7 years for every 10% increase in family members affected with diabetes.^28^ A similar finding was observed in a recent study from India, where 69% of those diagnosed at ≤40 years had a family history of diabetes.^11^

We had earlier reported that there is a rising trend of T2DM in the low and middle socioeconomic groups.^7^ In this analysis, we noted rural habitat to have an effect on diabetes in the older population. This could be due to unawareness, low socioeconomic status, and inhibition to seek medical support. Moreover, in our previous study,^32^ we reported that prevalence of cardiovascular risk factors rapidly increased among the rural habitants, particularly in women.^32^

Early progression to T2DM is also attributed to adverse lifestyle factors where less physical activity and consumption of energy‐rich foods contribute to increased adiposity resulting in insulin resistance and chronic inflammation.^33^ Using the 24‐hour recall dietary assessment, we observed that the total calorie consumption increased in the population over a 10‐year period, but was similar in both age categories. The proportion having heavy physical activity significantly reduced with an increase in sedentary behavior in these individuals. Among the T2DM cases, changes in physical activity were noted only among the younger group. A greater proportion had light activity; persons with sedentary behavior increased while those having heavy activity decreased over the study period.

There is emerging evidence from recent studies that individuals diagnosed at age <40 years have reduced insulin sensitivity and insulin secretory capacity than in people who develop T2DM later in life.^11^, ^34^ This deterioration of beta‐cell function may be closely associated with the severity of insulin resistance due to increased visceral adiposity, chronic low‐grade inflammation, or genetic determinants. Asian Indian populations could be insulin resistant even without overt obesity.^6^ A recent study showed that among the individuals with normal BMI, the prevalence of T2DM at <40 years was 9.3% in the white Europeans, whereas it was 24%–39% among the Asian Indians.^11^ Analysis of the clinical data and process‐specific polygenic score showed that onset of T2DM at younger ages was associated with genetically determined lower beta‐cell function than individuals with older onset.^11^

Early mortality had been reported among younger individuals with T2DM due to faster metabolic deterioration and the risk of premature vascular complications.^35^ Data from the Swedish National Registry showed that compared with age and sex‐matched individuals in the general population, the relative risks of all‐cause mortality (hazard ratio [HR] = 2.05, 1.81–2.33), coronary heart disease (HR = 4.33, 3.82–4.91), acute myocardial infection (HR = 3.41, 2.88–4.04), congestive heart failure (HR = 4.77, 3.86–5.89), and stroke (HR = 3.58, 2.97–4.32) were higher for individuals with T2DM diagnosed ≤40 years of age, than that of individuals diagnosed above this age. These risks attenuated progressively with each decade increase in age of diagnosis.^36^ Similar findings reported from other countries were suggestive that long‐term hyperglycemia and suboptimal management of diabetes lead to high prevalence of secondary complications in the younger population.^37^, ^38^

The main highlight of our study is, to the best of our knowledge, it may be the first population based data to report the prevalence and incidence among persons aged 20–39 years and ≥40 years, 10 years apart using similar methodology. Previous studies that had used clinic‐ or hospital‐based data probably could have overestimated the prevalence rates. We observed that in both age categories, the ratio of KDM to NDM decreased, indicating that this was not because of higher detection rates during screening; the proportion of incident cases to total KDM rose significantly, indicative that higher incidence could be a recent phenomenon in the population. The possible limitations were that, in the two surveys, numbers recruited in the younger category was less reflecting a smaller proportion in the overall sample. The prevalence of complications could not be recorded, and beta‐cell function and insulin resistance were not assessed.

The increasing number of T2DM in the younger population is a cause of concern particularly in a low‐ and middle‐income country like India where more than 74.2 million live with diabetes.^1^ Recent reports from a national survey concluded that the prevalence of diabetes and prediabetes in India is 11.4% (95% CI, 10.2–12.5) and 15.3% (95% CI, 13.9–16.6), respectively.^39^ The incidence of T2DM is also on the increase particularly in the urban and semiurban settings of Tamil Nadu, India, indicating adverse societal influences.^40^ We now report that the increase is not only in the older population but also among the youth. These changes could be due to increased parental history of diabetes and prevalence of obesity in particular abdominal obesity in the younger population. Diabetes in the young invariably leads to more years of disease burden, early onset of complications and increased healthcare costs. In accordance with an earlier publication,^41^ our findings also emphasize the need for early detection and prevention in Asian Indians through screening and risk stratification.

## AUTHOR CONTRIBUTIONS

AR is the Principal Investigator; AR, CS, AN, Arun R contributed to the study design and developed the protocol. KS and PS coordinated the fieldwork, data collection, and supervised the conduct of the study. AR, CS, AN, KS, and PS contributed to data preparation and analysis. AR, CS, AN, PS, KS, and Arun R drafted the manuscript and revised it with critical input. All authors have read and approved the final draft.

## FUNDING INFORMATION

The study was funded by India Diabetes Research Foundation, Chennai.

## CONFLICT OF INTEREST STATEMENT

The authors declare no conflicts of interest.

## PATIENT CONSENT STATEMENT

Written informed consent was obtained from all enrolled participants prior to start of the study procedures.

## CLINICAL TRIAL REGISTRATION

The primary study was registered with www.ClinicalTrials.gov. Identifier: NCT03490136.

## Data Availability

Data are available with the corresponding author and will be shared on reasonable request.
